# [Corrigendum] Succinate promotes skeletal muscle protein synthesis via Erk1/2 signaling pathway

**DOI:** 10.3892/mmr.2024.13293

**Published:** 2024-07-17

**Authors:** Yexian Yuan, Yaqiong Xu, Jingren Xu, Bingqing Liang, Xingcai Cai, Canjun Zhu, Lina Wang, Songbo Wang, Xiaotong Zhu, Ping Gao, Xiuqi Wang, Yongliang Zhang, Qingyan Jiang, Gang Shu

Mol Med Rep 16: 7361–7366, 2017; DOI: 10.3892/mmr.2017.7554

Following the publication of the above article, the authors realized that, in Fig. 1D on p. 7363, the data panel selected for the ‘0.5 mM Succinate’ group was duplicated in Fig. 1B (Control) in another article of theirs published in *FASEB J* (“α-Ketoglutarate prevents skeletal muscle protein degradation and muscle atrophy through PHD3/ADRB2 pathway”: doi: 10.1096/fj.201700670R) due to the fact that they had inadvertently confused the layout of the two figures. The authors apologize for this error. Secondly, in terms of the quantification of the blots shown in Fig. 2A, β-actin was not in fact used as a loading control; the phosphoproteins were normalized against the levels of the relative total protein, and the layout of Fig. 2A has been revised to reflect this (note that the the figure legend for Fig. 2 has also been revised: The last sentence no longer reads, “β-actin was used as a loading control.”).

The revised versions of Figs. 1 and 2 are shown on the next page. Note that these errors did not affect the results or the main conclusions reported in the study, and no corrections were required either to the descriptions in the text or to the histograms shown in these figures. All the authors approve of the publication of this corrigendum, and the authors are grateful to the Editor of *Molecular Medicine Reports* for allowing them the opportunity to publish this. The authors regret their oversight in allowing these errors to be included in the paper, and apologize to the readership for any inconvenience caused.

## Figures and Tables

**Figure 1.**
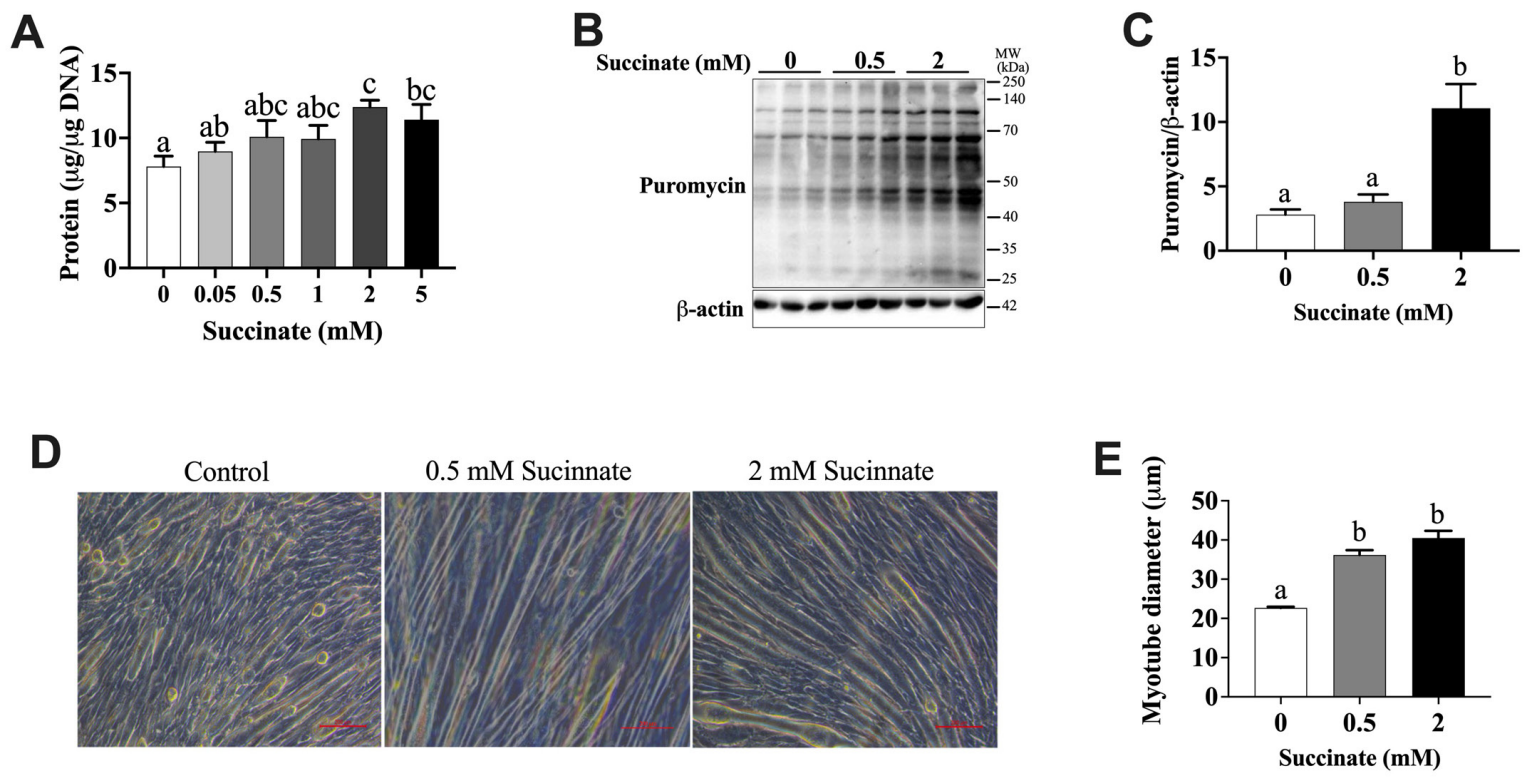
Succinate dose-dependently promoted protein synthesis in C2C12 myotubes. C2C12 cells were cultured in a differentiation medium for 6 days. Then C2C12 myotubes were treated with different concentrations of succinate (0, 0.05, 0.5, 1, 2 and 5 mM) for 48 h. (A) Total protein levels. (B) Puromycin incorporation detected by western blotting. (C) The statistical analyses result of the western blotting of the Puromycin. (D) The morphology of C2C12 myotubes. (E) The statistical analyses result of the C2C12 myotubes diameter. Data are presented as mean ± SEM. ^a-c^Significant differences between groups (P<0.05). β-actin served as a loading control.

**Figure 2.**
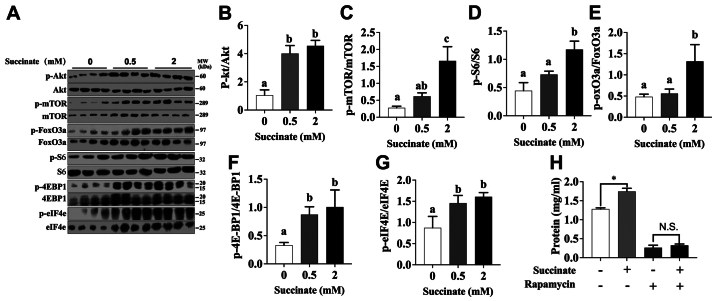
Akt/mTOR/FoxO cascade was involved in succinate-induced protein deposition in C2C12 myotubes. C2C12 cells were cultured for 6 days in a differentiation medium. C2C12 myotubes were then exposed to succinate (0.5 and 2 mM) for 48 h. (A) Western blot analysis of p-Akt, Akt, p-mTOR, mTOR, p-FoxO3a, FoxO3a, p-S6, S6, p-4EBP1, 4EBP1, p-eIF4e and eIF4e. (B-G) The statistical analyses results of the western blotting of the phosphorylation level of Akt, mTOR, FoxO3a, S6, 4E-BP1 and eIF4e. (H) The total protein level after C2C12 cells were co-treated with succinate and rapamycin. ^a,b^Significant differences between groups (P<0.05). *P<0.05 compared with the control.

